# Polarizing Perspectives: Ion- and Dipole-Induced Dipole Interactions Dictate Bulk Nanobubble Stability

**DOI:** 10.1021/acs.jpcb.4c03973

**Published:** 2024-07-11

**Authors:** Mohammadjavad Karimi, Gholamabbas Parsafar, Hamidreza Samouei

**Affiliations:** Department of Petroleum Engineering, Texas A&M University, College Station, Texas 77843, United States

## Abstract

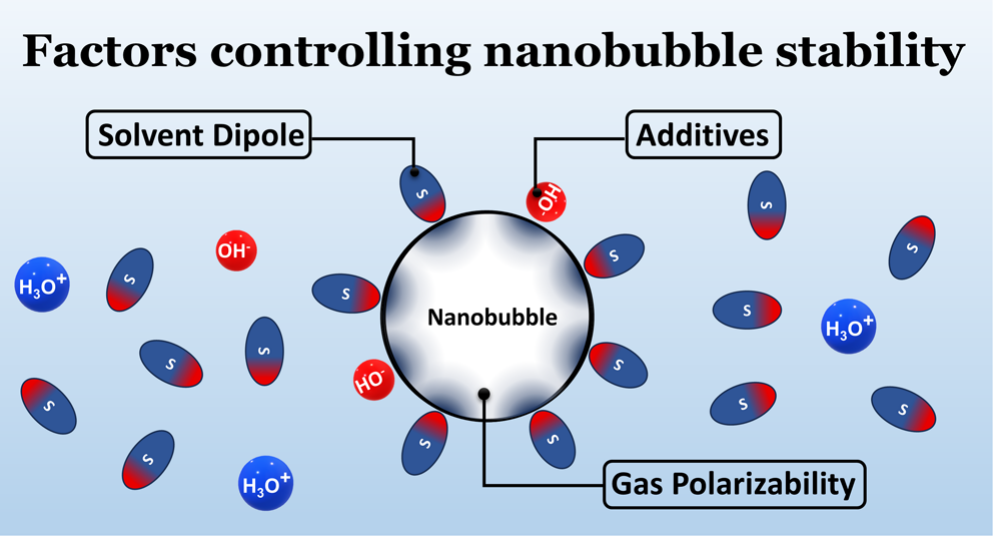

The origin of the stability of bulk Nanobubbles (NBs) has been the object of scrutiny in recent years. The interplay between the surface charge on the NBs and the Laplace pressure resulting from the surface tension at the solvent-NB interface has often been evoked to explain the stability of the dispersed NBs. While the Laplace pressure is well understood in the community, the nature of the surface charge on the NBs has remained obscure. In this work, we aim to show that the solvent and the present ions can effectively polarize the NB surface by inducing a dipole moment, which in turn controls the NB stability. We show that the polarizability of the dispersed gas and the polarity of the dispersing solvent control the dipole-induced dipole interactions between the solvent and the NBs, and that, in turn, determines their stability in solution.

## Introduction

Bulk nanobubbles (NBs) are a class of materials that consist of bubbles with dimensions on the nanometer scale that have captured the interest of scientists and engineers from a variety of disciplines.^1−4^ These materials have found application in an assortment of areas, such as water treatment and disinfection,^5−9^ agriculture and hydroponics,^10−15^ and most recently, advanced chemical oxidation processes.^16−20^ Unlike bubbles of larger diameter (>1 μm), NBs are known to be stable in water for long periods of time.^21−25^ The standard model currently adopted in the literature to explain this stability involves a balance between the Laplace pressure and the pressure from electrostatic interactions at the NB-solvent interface. In general, we can express this balance using the Young–Laplace equation as follows:
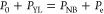
1where *P*_0_ is the pressure of the atmosphere outside the solution, *P*_YL_ is the Laplace pressure, *P*_NB_ is the pressure inside the NB, and *P*_e_ is the electrostatic pressure. If *P*_e_ is neglected, then the internal pressure of NBs must be unreasonably high to counteract the effects of surface tension.^1,24^ For example, if we substitute γ = 0.072 N/m for the surface tension of pure water, and *r* = 1.0 × 10^–7^ m for the radius of the NB into the Laplace equation,
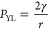
2we get *P*_YL_ = 14.4 bar and *P*_NB_ = 15.4 bar for a bubble with a radius of 100 nm. Therefore, several researchers have sought alternative conceptual frameworks to explain the stability of NBs. However, many of them are very specific to the bubble generation circumstances and lack generalizability, and many of them make imprecise predictions on the properties of NBs. Ljunggren and Eriksson pioneered a theoretical model for the stability of colloidal gas particles that predicted their lifetimes to be 1–100 μs,^26^ while NBs in water have been reported to be stable up to a few months.^21−25^ Attard et al. has also been unsuccessful in explaining the stability of NBs without invoking electrostatic effects.^27,28^

**Figure 1:**
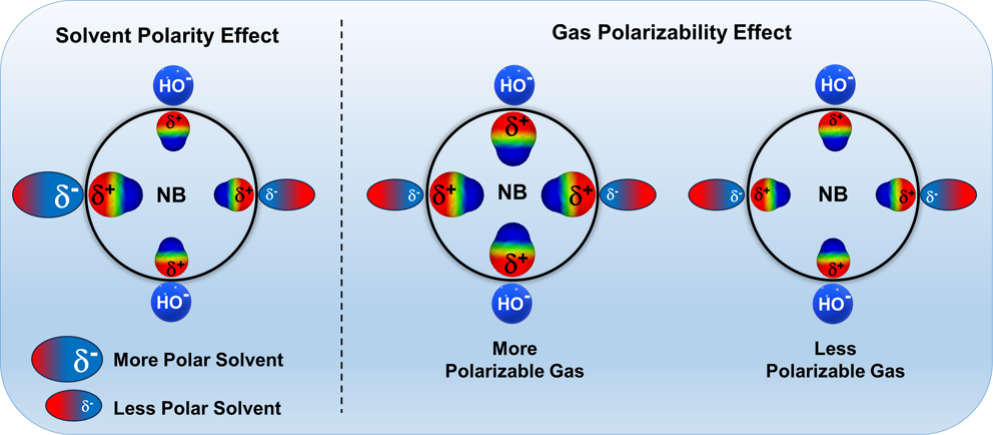
Effect of solvent polarity (left panel), gas polarizability (right panel), and additives (OH^–^) on the dipole induced on nanobubbles. The dumbbells represent the dipole moment induced on the NB, and dumbbell sizes are proportional to the magnitude of the induced dipole.

Seddon et al. proposed the hypothesis that NBs are stabilized by the “presence of adsorbed material”.^29^ While some reports have pointed to the possibility that nanobubbles could potentially be confused with impurities in the solution,^30,31^ many others clearly demonstrate the existence of bulk NBs in pure water.^22,23,32−38^ It has been elucidated that additives such as surfactants^33,39^ and electrolytes^21,40^ affect NB size distribution, zeta potential, and stability. However, the concentration of such species in pure deionized water is likely too low to stabilize bulk NBs sufficiently.^31^ In line with the proposition that hydroxide ions from autoionization of pure water at neutral pH impart stability onto NBs,^41−43^ Satpute and Earthman recently suggested a theoretical model based on the physisorption of hydroxide onto shrinking microbubbles that turn into NBs and stabilize them by electrostatic repulsions.^44^ This theory has taken the experimentally observed negative partial charge on the NB surfaces into account^41,45^ and has made significant conceptual progress with respect to its predecessors. However, it suffers from various shortcomings. First, it can only be applied to NBs generated by ultrasonic cavitation; second, it assumes that the NBs result from shrinking microbubbles; and third, it only applies to aqueous solutions. Additionally, it has been established in the literature that at nanometer scale, higher curvature terms in the Young–Laplace equation become noticeable, albeit in the hundreds of nanometer scale, these higher order terms do not seem to have a major effect on the surface tension at the water-NB interface.^46^ These considerations have swayed researchers toward hypothesizing that bulk NBs could be stabilized by electrostatic interactions (*P*_e_ in eq 1). While Wang et al. recently suggested that such interactions may not be strong enough to abate the Laplace pressure,^47^ Koshoridze et al. argued that from a theoretical standpoint, *P*_e_ could be large enough to impart substantial stability to NBs.^48^ Our objective in this work is to investigate this contention and provide and assess the validity of a more general theory that would address the aforementioned shortcomings and explain the stability of NBs. Expanding these considerations beyond the water and N_2_ NB system to a general solvent-NB manifold led us to develop the hypothesis that the electrostatic interactions between the NBs and their surrounding environment determine their stability. To test this hypothesis, we identify three main factors that control the behavior of this generalized system and assess their role independently. These factors are (i) the identity of the gas inside the NB (atomic or molecular polarizability of the gas), (ii) the nature of the dispersing solvent (solvent polarity), and (iii) the presence of additives (Figure 1).

## Experimental Methods

### Materials

All solvents and chemicals were used as received. Deionized water (measured conductivity, σ = 0.012 mS/cm, ACS reagent grade) was obtained from LabChem. Methanol (99%) and Ethanol (200 proof) were obtained from Koptec. Acetonitrile (99.9%) was purchased from Fisher Chemical. Hexanes (99%) was purchased from JT-Baker. KOH reagent grade was purchased from Sigma-Aldrich. Nitrogen (99.9%), oxygen (99.9%), helium (99.99%), *n*-butane (99%), and CO_2_ (99.9%) were used as received. Particle size analyses using NTA (Nanoparticle Tracking Analysis) were performed at 22 °C on a NanoSight LM10 equipped with a CMOS detector and a 405 nm violet laser light source. Each measurement consisted of five runs, each from a randomly selected part of the sample solution, and the presented data is the statistical average of the runs. Zeta potential measurements were carried out on a Malvern Zetasizer Nano ZS equipped with a 532 nm green laser. For each measurement, an optically transparent DTS 1070 cell was filled with the analyte solution and equilibrated at 20 °C and constant potential for 2 min before collecting the data. We note that accurate readings were only possible by carefully inputting the dielectric constant and viscosity of the solvent medium, as well as the refractive index of the dispersed gas in the Malvern software for each NB-solvent pair studied. The values implemented in this study are given in Tables 1(49−55) and 2.^56,57^ For NTA measurements, solvent viscosities given in Table 1 were implemented for accurate particle size distribution readings. For the case of organic solvents, a glass syringe was used for both the zeta potential and NTA measurements to avoid any possible contamination by particles detaching from plastic syringes. All experiments were performed in triplicate based on which errors were estimated.

**Table 1:** Solvent Viscosity and Dielectric Constants Used in This Study

solvent	dielectric constant	viscosity (cP)
water	80.37	1.003
methanol	32.66	0.55
4:1 water/methanol (*V*/*V*)	70.82	1.34
1:1 water/methanol (*V*/*V*)	56.51	1.54
1:4 water/methanol (*V*/*V*)	42.20	1.05
ethanol	25.07	1.19
4:1 water/ethanol (*V*/*V*)	71.23	1.81
1:1 water/ethanol (*V*/*V*)	54.97	3.08
1:4 water/ethanol (*V*/*V*)	36.81	2.37
acetonitrile	35.95	0.34
hexane	1.88	0.30

**Table 2:** Gas Refractive Indexes (*n*_D_) and Atomic or Molecular Polarizabilities (α) Used in This Study

gas	*n*_D_	α /Å^3^
He	1.000032	0.208
N_2_	1.000274	1.71
O_2_	1.000258	1.562
CO_2_	1.000434	2.507
*n*-butane	1.3326	8.02

Scanning Electron Microscopy (SEM) was conducted on a TESCAN Fera 3 instrument operating at 15 kV source voltage and 9 mm lens to detector distance. Images were collected on secondary electron mode.

### Nanobubble Generation

**Figure 2:**
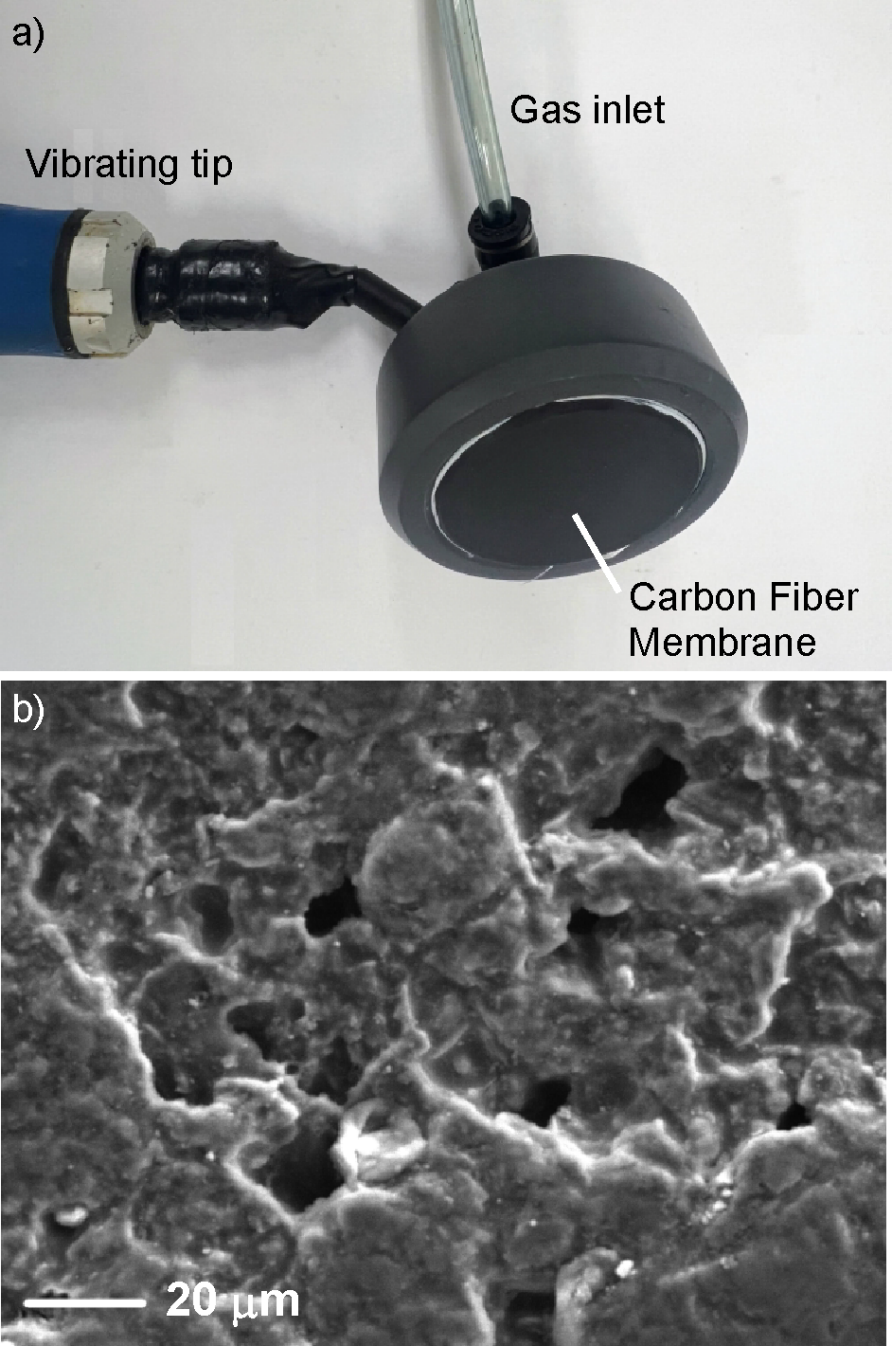
(a) Picture of the NB generator used in this work. (b) Secondary electron SEM image of the carbon fiber membrane incorporated in the NB generator.

Our method of generation consisted of diffusion of gas at a pressure of 30 psi (∼2 bar) through a porous carbon nanofiber membrane (*d* = 2–9 μm) immersed in the solvent at a gas flow rate of ∼10 mL/min similar to a previously reported method involving a ceramic membrane.^58^Figure 2 shows a picture of the NB generator used in this work (a) and an SEM image of the carbon fiber membrane implemented within it. The membrane was mounted on a rod and vibrated at ∼10 Hz throughout the generation period to help with the homogeneity of the NBs. The nanobubbler was custom-made by Environmental Compliance Equipment and used as purchased.

To a 500 mL glass beaker was added 250 mL of the solvent. The carbon fiber membrane mounted on a vibrating tip was connected to a tank of the gas of interest, kept at a stream of 30 psi, and then immersed in the solvent. The solution was thus sparged for 15 min; then, the vibrator was taken out and powered off. The position of the carbon fiber membrane is crucial to the reproducibility of the size and distribution of the nanobubbles. The membrane was held with a clamp exactly halfway down into the solution, and its back was positioned ∼1 cm away from the side wall of the beaker to provide as much space as possible for the nascent nanobubbles to travel in the solution without hitting the glass. The beaker was secured in place to prevent movement due to the vibrations. The presence of NBs was confirmed by running the generation protocol with the gas turned off, for which no suspended particles were observed at the detection limit of NTA (10^6^ mL^–1^).

## Results and Discussion

As a starting point, we focus on the case of N_2_ NBs in pure water. We consider that the negative zeta potential observed for N_2_ NBs in water (ζ = −20 – −29 mV)^41,59,60^ stems from the oxygen atoms of water and hydroxide anions orientating toward and polarizing the surface of the NBs, as those are the only species capable of inducing a dipole. We note that negatively charged hydroxide ions are more potent than neutral water molecules at inducing a dipole on the NB surface. This means that while the polarizing effect of water molecules on the zeta potential of the NBs may not be negligible, the main contribution originates from hydroxide anions. This induced dipole may be somewhat counterbalanced by the H_3_O^+^ ions. However, the charge density on hydroxide anions is much greater than that on hydronium cations, as hydronium ions are known to form larger clusters with the nearby water molecules, effectively dissipating the positive charge, whereas such a phenomenon has not been known for hydroxide ions.^61,62^ This means that hydronium cations are less effective than hydroxide anions at inducing a charge on the NB surface, and by means of replacing the more stabilizing hydroxide ions at lower pH, they may have a detrimental effect on NB stability. Indeed, this is corroborated by previous studies showing that NBs are much less stable under acidic conditions than in neutral to basic solutions.^25,59,63^

**Figure 3:**
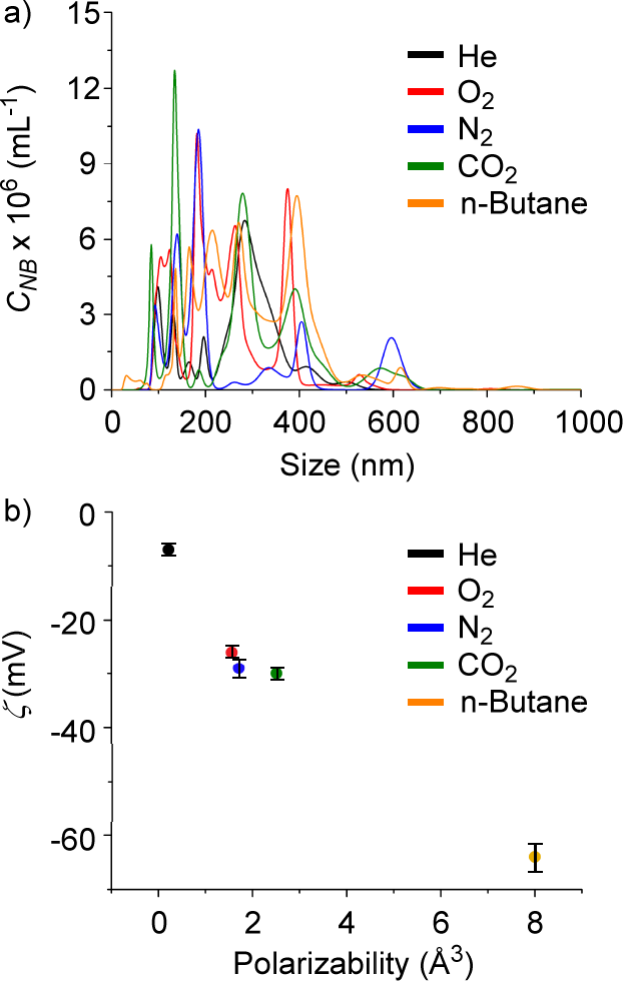
(a) NTA analysis showing particle size distribution profile of the NBs generated in water using different gases. (b) Plot of zeta potential measured after 1 h on aqueous solutions of the gases as a function of gas polarizability. The polarizability values were adapted from the NIST database.^57^

In our proposed model, the solvent and any ions or additives that may be present induce a dipole on the NB surface by dipole-induced dipole or ion-induced dipole interactions. We also know from the theory of colloidal stability developed by Derjaguin, Landau, Verwey, and Overbeek (DLVO) that the magnitude of the induced surface charge or the zeta potential directly controls the colloidal stability of monodisperse systems.^59,64^ We can hypothesize that the zeta potential and the stability of the NBs are determined by how well they accommodate the dipole induced by their environment. This is best reflected in the polarizability of the gas molecules or atoms (α), which is a measure of how easily an external electric field can perturb the electron density of the gas atom or molecule and, therefore, reflects the ability of the gas molecules to accommodate surface charge. To assess this hypothesis, we decided to study aqueous NBs of five different gases with a wide range of α values. Analysis of the size distribution and number density of the resulting solutions by NTA shows that for all the gases studied, most of the bubbles have a diameter of 100–500 nm 1 h after generation (Figure 3a). The bubble number densities were in the range of 8.2 × 10^7^ – 1.3 × 10^8^ bubbles/mL, with the least polarizable gas Helium (α = 0.2 Å^3^) lying on the lower end and the highly polarizable *n*-butane (α = 8.0 Å^3^) on the higher end of the range, respectively. The size distribution profile for the individual solutions displayed in Figure 3a are given in the Supporting Information (Figures S1–S5). For the control experiment, we turned the gas off and repeated the NB generation procedure, for which we observed no suspended particles. This provides strong evidence that the nanoentities observed by NTA during NB generation with the gas on are in fact NBs.

We then subjected these solutions to zeta potential analysis. To our delight, we found a direct correlation between the zeta potential of the NBs and the polarizability of the gas (Figure 3b), with helium showing the lowest number ζ_He_ = −7.3 ± 2.1 mV and *n*-butane showing a remarkably high zeta potential of ζ_Bu_ = −65.4 ± 5.6 mV. It is worth noting that some studies report that ζ_N2_ is less negative than ζ_O2_ in water.^41,65^ Our measurements, however, in agreement with other reports,^40,58^ show the opposite relationship with −26.0 ± 1.8 mV for O_2_ and −29.1 ± 2.0 mV for N_2_. The zeta potential distribution for all five gases studied here are given in Figures S6–S10.

The order of the atomic or molecular polarizabilities (α) in these gases is He < O_2_ < N_2_ < CO_2_ < *n*-butane. If our hypothesis that NB stability is controlled by α was correct, we would expect the same trend for the order of the stability of the NBs of these gases. Indeed, NTA analysis of the same solutions 1 week after NB generation confirms this notion (Figures S1–S5). The steepest decline in number density was observed for the least polarizable gas Helium with a 47% decrease, followed by O_2_ with 14%, and N_2_ with 11%. The highest stability was observed for the highly polarizable and virtually unaltered *n*-butane that conserved 99% of the bubbles. In line with previous reports on CO_2_ NBs,^12,40,66−72^ these bubbles proved to be unstable as almost no NBs remained after 1 day (Figure S4), and the initial pH of the NB solution at 5.3 rose to 7.2, suggesting that the CO_2_ gas had escaped the solution. This unique behavior may be explained by considering that the CO_2_ molecules inside the NBs can gradually escape the solution via the dynamic equilibria involving their dissolution to form CO_2_ (aq), H_2_CO_3,_ and HCO_3_^–^, thus providing a very kinetically facile mechanism for their release to the atmosphere.^40,70^ This may also be exacerbated by the physisorbtion of hydroxide ions on the surface of CO_2_ NBs, facilitating the rapid removal of the gas molecules by hydroxide ions from the nanobubble and their dissolution into the solution as HCO_3_^–^ ions. Such gas reactivity arguments have also been recently proposed for the electrochemical reduction of O_2_ in H_2_O_2_ production systems.^73^ These results, taken together, showcase the validity of the hypothesis that the polarizability of the NB gas plays a crucial role in its stability by accommodating the dipole induced by the solvent.

**Figure 4:**
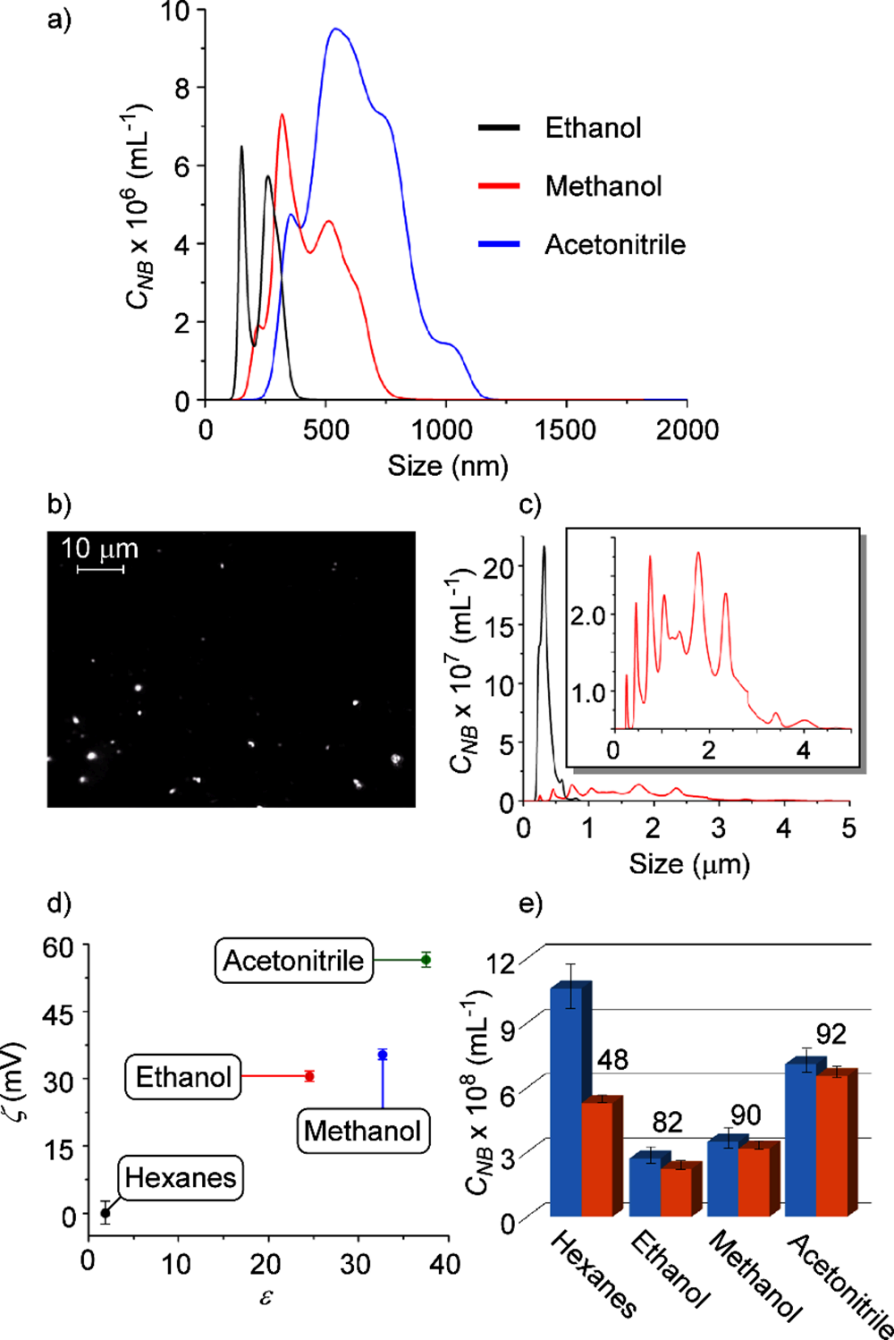
(a) NTA analysis of N_2_ NBs generated in polar organic solvents showing the particle size distribution profile 1 h after NB generation. (b) NTA micrograph of N_2_ NBs generated in hexanes after 1 h. (c) NTA analysis of N_2_ NBs generated in hexanes showing the particle size distribution profile. The black trace shows the distribution 1 h after generation and the red trace shows the distribution after 1 day. The inset shows an expansion of the particle size distribution after 1 day. (d) Zeta potential of N_2_ NBs in different organic solvents as a function of the dielectric constant of the solvent. (e) Particle number density of N_2_ NBs in different organic solvents 1 h (blue columns) and 24 h (red columns) after generation. The numbers on the graph are the percentages of the bubbles remaining in the solution after 24 h.

Given that the core of our proposed conceptual framework involves ion- and dipole-induced dipole interactions between the solvent and the present ions with the dispersed NBs, we then questioned whether the polarity of the solvent controls the magnitude of the induced dipole and the zeta potential, and by means of that, the stability of NBs. To answer this question, we decided to study the zeta potential of dispersed N_2_ NBs in five solvents of different polarity and dielectric constant (ε), namely water, methanol, ethanol, acetonitrile, and hexanes. Our choice of solvent was limited by compatibility with the zeta potential cell, as certain solvents turned the cell walls opaque and rendered Dynamic Light Scattering (DLS) measurements impossible. We first confirmed the successful generation of N_2_ NBs by NTA, and compared the results to those obtained on control solutions that were subjected to the NB generation procedure with the gas turned off, for which we observed no suspended particles. In all cases, the bubble number density was in the range of 2.2 × 10^8^ – 1.1 × 10^9^ mL^–1^, and for the case of the polar solvents, the bubble size distributions show the rather polydisperse nature of the NBs (Figure 4a).

Hexanes, however, displayed a unique behavior. Figure 4b shows an NTA micrograph of the N_2_ NBs suspended in hexanes. Unlike the other solvents studied, the size distribution in this system was narrow (Figure 4c), with a mean particle size of 340 nm 1 h after NB generation. This distribution underwent drastic changes over time, and after 24 h, the smaller bubbles had almost completely vanished, and larger bubbles with a mean diameter of 1663 nm emerged in the solution along with microbubbles as large as 4000 nm (Figure 4c, inset). These changes in size distribution were accompanied by a stark decrease in the bubble number density from 1.1 ± 0.2 × 10^9^ mL^–1^ down to 5.3 ± 0.3 × 10^8^ mL^–1^, and no particles were observed in this solvent after 1 week. Taken together, it becomes clear that over time, the smaller bubbles formed during the generation process merge to form larger bubbles in hexanes. This can be explained by the fact that hexanes cannot impart colloidal stability due to its highly nonpolar nature, thus allowing the NBs that come in proximity to one another to merge, forming larger bubbles that rise to the surface and burst. The absence of any charged particles observed by zeta potential measurement on this solution further supports this point and provides strong evidence that electrostatic interactions are vital to NB stability.

Further bolstering this view, zeta potential analysis on the other organic solutions shows a direct correlation between the dielectric constant of the solvent and the magnitude of the measured zeta potential (Figure 4d). This is consistent with our hypothesis that NBs are stabilized by dipole-induced dipole interactions, as the larger dielectric constant directly translates to a stronger ability to induce a dipole on the NB surface, leading to a higher zeta potential.

The positive sign of the potentials could be rationalized by the lack of water molecules and, therefore, hydroxide ions in these solutions. In fact, when N_2_ NBs were generated in a 1.0 × 10^–4^ M solution of KOH in ethanol, a zeta potential of −5.5 ± 1.9 mV was observed, whereas the zeta potential in pure ethanol was 30.2 ± 3.8 mV. A similar result was obtained when deliberately 3.6 μL of a saturated solution of KOH in ethanol (∼7 M) was added to NBs generated in pure ethanol to reach a similar concentration of hydroxide. This highlights the effect of additives on the zeta potential of NBs, and further evidences the importance of electrostatic interactions on NB stability. In line with these arguments, NTA analysis of the organic solutions after 1 day (Figures S11–S13) shows that the bubble number density undergoes a more pronounced drop as the solvent polarity decreases (Figure 4e). These results indicate that the dielectric constant of the solvent, which is a direct measure of its polarity and the magnitude of the permanent dipole moment of its molecules, controls the stability of NBs. This in turn signifies that the main stabilizing effect of the solvent is by interacting with the NBs through dipole-induced dipole interactions.

Attempting to shed more light on the nature of the solvent-NB and additive-NB interactions, we then decided to study NBs in aqueous–organic solvent mixtures. We prepared solutions of N_2_ NBs in water–ethanol^36,71,74,75^ and water–methanol media of varying compositions. Although in previous studies low zeta potentials around ±5 mV have been observed for simple alcohols,^76,77^ those studies may not have carefully considered the effects of solvent viscosity and dielectric constant, as well as the refractive index of the gas on the zeta potential (Tables 1 and 2, vide supra). Moreover, in most of these studies the bubble number densities are on the order of 10^6^ – 10^7^ mL^–1^, which may be too low to allow for reliable zeta potential measurements. Additionally, it has been shown that nanobubbles may form by simply mixing water with alcohols.^75,78,79^ We also do observe NBs by mixing methanol and ethanol with water. However, the number density of these bubbles is on the order of 10^6^ – 10^7^ mL^–1^, which is at least an order of magnitude lower than in our mixtures after NB generation.

**Figure 5:**
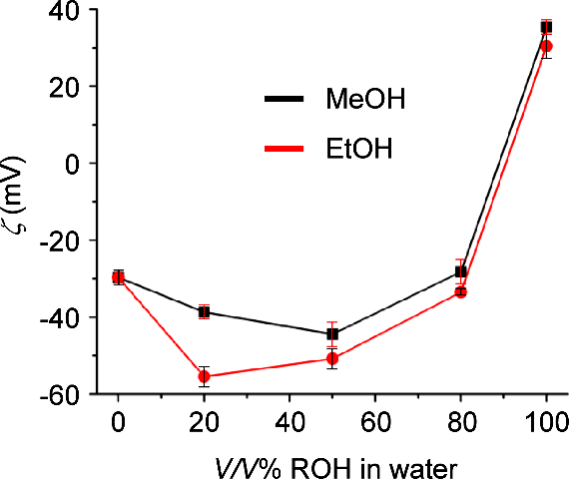
Zeta potential of N_2_ NBs in water alcohol mixtures at different compositions.

For both water–ethanol and water–methanol, the measured potentials are more negative than in pure water at intermediate water to alcohol volumetric ratios (Figure 5). This trend suggests that at such ratios, hydroxide ions are more strongly adsorbed on the NB surface, and polarize the interface much more effectively. These results could be further rationalized by considering that the solvent medium can compete with the NBs for the hydroxide ions, including the ones adsorbed on the NB surface. This phenomenon is analogous to that established in Lewis acid chemistry, where a lower anion affinity for fluoride and hydroxide is observed in more polar solvents.^80,81^ In line with this, the zeta potentials measured at intermediate water:methanol ratios are lower in magnitude than those in similar water:ethanol compositions, as methanol is more polar than ethanol and by means of stronger hydrogen bonding with hydroxide ions, it provides more competition for the hydroxide anions adsorbed on the NB surface. At lower water content, the stronger adsorption is offset by the opposing effect of lower availability of hydroxide ions due to the smaller amount of water in the solution, and the zeta potential becomes less negative. These results showcase the effect of solvent on the adsorption of ions on NB surfaces, controlling their colloidal stability.

## Conclusions

In conclusion, we provide a new conceptual framework to rationalize the stability of NBs based on the ion- and dipole–induced dipole interactions between the solvent and the NB, as well as the effect of additives. We explored the viability of this theory by showing that NB stability is controlled by the polarity of the solvent, the magnitude of the induced dipole on the NB gas, and the adsorption of additive ions. The interplay between these three factors determines the potential at the slipping plane of the NBs and controls the probability of their combination upon coming in proximity. This work aims to enhance our fundamental understanding of NBs and paves the way for their implementation in gas storage and utilization systems, such as CO_2_ sequestration and O_2_ reduction.

## Supporting Information Available

Additional particle size analysis and zeta potential data (PDF)

## References

[ref1] Alheshibri M.; Qian J.; Jehannin M.; Craig V. S. A History of Nanobubbles. Langmuir 2016, 32, 11086–11100. 10.1021/acs.langmuir.6b02489. 27594543

[ref2] Zhou L.; Wang S.; Zhang L.; Hu J. Generation and stability of bulk nanobubbles: A review and perspective. Curr. Opin. Colloid Interface Sci. 2021, 53, 101439 10.1016/j.cocis.2021.101439.

[ref3] Foudas A. W.; Kosheleva R. I.; Favvas E. P.; Kostoglou M.; Mitropoulos A. C.; Kyzas G. Z. Fundamentals and applications of nanobubbles: A review. Chem. Eng. Res. Des. 2023, 189, 64–86. 10.1016/j.cherd.2022.11.013.

[ref4] Marcelino K. R.; Ling L.; Wongkiew S.; Nhan H. T.; Surendra K. C.; Shitanaka T.; Lu H.; Khanal S. K. Nanobubble technology applications in environmental and agricultural systems: Opportunities and challenges. Crit. Rev. Environ. Sci. Technol. 2023, 53, 1378–1403. 10.1080/10643389.2022.2136931.

[ref5] Atkinson A. J.; Apul O. G.; Schneider O.; Garcia-Segura S.; Westerhoff P. Nanobubble Technologies Offer Opportunities To Improve Water Treatment. Acc. Chem. Res. 2019, 52, 1196–1205. 10.1021/acs.accounts.8b00606. 30958672

[ref6] Khan P.; Zhu W.; Huang F.; Gao W.; Khan N. A. Micro–nanobubble technology and water-related application. Water Supply 2020, 20, 2021–2035. 10.2166/ws.2020.121.

[ref7] Wu J.; Zhang K.; Cen C.; Wu X.; Mao R.; Zheng Y. Role of bulk nanobubbles in removing organic pollutants in wastewater treatment. AMB Express 2021, 11, 96–108. 10.1186/s13568-021-01254-0. 34184137 PMC8239109

[ref8] Fujita T.; Kurokawa H.; Han Z.; Zhou Y.; Matsui H.; Ponou J.; Dodbiba G.; He C.; Wei Y. Free radical degradation in aqueous solution by blowing hydrogen and carbon dioxide nanobubbles. Sci. Rep. 2021, 11, 3068 10.1038/s41598-021-82717-z. 33542381 PMC7862495

[ref9] Jia M.; Farid M. U.; Kharraz J. A.; Kumar N. M.; Chopra S. S.; Jang A.; Chew J.; Khanal S. K.; Chen G.; An A. K. Nanobubbles in water and wastewater treatment systems: Small bubbles making big difference. Water Res. 2023, 245, 120613 10.1016/j.watres.2023.120613. 37738940

[ref10] Zhang H.; Lyu T.; Bi L.; Tempero G.; Hamilton D. P.; Pan G. Combating hypoxia/anoxia at sediment-water interfaces: A preliminary study of oxygen nanobubble modified clay materials. Sci. Total Environ. 2018, 637–638, 550–560. 10.1016/j.scitotenv.2018.04.284. 29754089

[ref11] Wang Y.; Wang S.; Sun J.; Dai H.; Zhang B.; Xiang W.; Hu Z.; Li P.; Yang J.; Zhang W. Nanobubbles promote nutrient utilization and plant growth in rice by upregulating nutrient uptake genes and stimulating growth hormone production. Sci. Total Environ. 2021, 800, 149627 10.1016/j.scitotenv.2021.149627. 34426308

[ref12] Pal P.; Anantharaman H. CO_2_ nanobubbles utility for enhanced plant growth and productivity: Recent advances in agriculture. J. CO2 Util. 2022, 61, 102008 10.1016/j.jcou.2022.102008.

[ref13] Xue S.; Marhaba T.; Zhang W. Nanobubble Watering Affects Nutrient Release and Soil Characteristics. ACS Agric. Sci. Technol. 2022, 2, 453–461. 10.1021/acsagscitech.1c00238.

[ref14] Xue S.; Gao J.; Liu C.; Marhaba T.; Zhang W. Unveiling the potential of nanobubbles in water: Impacts on tomato’s early growth and soil properties. Sci. Total Environ. 2023, 903, 166499 10.1016/j.scitotenv.2023.166499. 37634716

[ref15] Huang M.; Nhung N. T. H.; Wu Y.; He C.; Wang K.; Yang S.; Kurokawa H.; Matsui H.; Dodbiba G.; Fujita T. Different nanobubbles mitigate cadmium toxicity and accumulation of rice (Oryza sativa L.) seedlings in hydroponic cultures. Chemosphere 2023, 312, 137250 10.1016/j.chemosphere.2022.137250. 36423719

[ref16] Wang B.; Wang L.; Cen W.; Lyu T.; Jarvis P.; Zhang Y.; Zhang Y.; Han Y.; Wang L.; Pan G.; Zhang K.; Fan W. Exploring a chemical input free advanced oxidation process based on nanobubble technology to treat organic micropollutants. Environ. Pollut. 2023, 340, 122877 10.1016/j.envpol.2023.122877. 37931673

[ref17] Hutagalung S. S.; Rafryanto A. F.; Sun W.; Juliasih N.; Aditia S.; Jiang J.; Arramel; Dipojono H. K.; Suhardi S. H.; Rochman N. T.; Kurniadi D. Combination of ozone-based advanced oxidation process and nanobubbles generation toward textile wastewater recovery. Front. Environ. Sci. 2023, 11, 1154739 10.3389/fenvs.2023.1154739.

[ref18] Ponce-Robles L.; Pagán-Muñoz A.; Lara-Guillén A. J.; Masdemont-Hernández B.; Munuera-Pérez T.; Nortes-Tortosa P. A.; Alarcón-Cabañero J. J. Full-Scale O3/Micro-Nano Bubbles System Based Advanced Oxidation as Alternative Tertiary Treatment in WWTP Effluents. Catalysts 2023, 13, 188–205. 10.3390/catal13010188.

[ref19] Li T.; Cui Z.; Sun J.; Li Q.; Wang Y.; Li G. Oxidative Capacity of Oxygen Nanobubbles and Their Mechanism for the Catalytic Oxidation of Ferrous Ions with Copper as a Catalyst in Sulfuric Acid Medium. Langmuir 2023, 39, 10112–10121. 10.1021/acs.langmuir.3c01047. 37452782

[ref20] Han W.-R.; Wang W.-L.; Qiao T.-J.; Wang W.; Su H.; Xu C.-X.; Wu Q.-Y. Ozone micro-bubble aeration using the ceramic ultrafiltration membrane with superior oxidation performance for 2, 4-D elimination. Water Res. 2023, 237, 119952 10.1016/j.watres.2023.119952. 37104935

[ref21] Bunkin N. F.; Shkirin A. V.; Ignatiev P. S.; Chaikov L. L.; Burkhanov I. S.; Starosvetskij A. V. Nanobubble clusters of dissolved gas in aqueous solutions of electrolyte. I. Experimental proof. J. Chem. Phys. 2012, 137, 054706 10.1063/1.4739528. 22894370

[ref22] Tanaka S.; Terasaka K.; Fujioka S. Generation and Long-Term Stability of Ultrafine Bubbles in Water *Chem*. Ing. Technol. 2021, 93, 168–179. 10.1002/cite.202000143.

[ref23] Agarwal K.; Trivedi M.; Nirmalkar N. Does salting-out effect nucleate nanobubbles in water: Spontaneous nucleation?. Ultrason. Sonochem. 2022, 82, 105860 10.1016/j.ultsonch.2021.105860. 34915251 PMC8683758

[ref24] Shi X.; Xue S.; Marhaba T.; Zhang W. Probing Internal Pressures and Long-Term Stability of Nanobubbles in Water. Langmuir 2021, 37, 2514–2522. 10.1021/acs.langmuir.0c03574. 33538170

[ref25] Montazeri S. M.; Kalogerakis N.; Kolliopoulos G. Effect of chemical species and temperature on the stability of air nanobubbles. Sci. Rep. 2023, 13, 16716 10.1038/s41598-023-43803-6. 37794127 PMC10550960

[ref26] Ljunggren S.; Eriksson J. C. The lifetime of a colloid-sized gas bubble in water and the cause of the hydrophobic attraction. Colloids Surf. A Physicochem. 1997, 129–130, 151–155. 10.1016/S0927-7757(97)00033-2.

[ref27] Attard P.; Moody M. P.; Tyrrell J. W. G. Nanobubbles: the big picture. Physica A 2002, 314, 696–705. 10.1016/S0378-4371(02)01191-3.

[ref28] Attard P. The stability of nanobubbles. Eur. Phys. J. Spec. Top. 2014, 223, 893–914. 10.1140/epjst/e2013-01817-0.

[ref29] Seddon J. R. T.; Lohse D.; Ducker W. A.; Craig V. S. J. A Deliberation on Nanobubbles at Surfaces and in Bulk. ChemPhysChem 2012, 13, 2179–2187. 10.1002/cphc.201100900. 22378608

[ref30] Häbich A.; Ducker W.; Dunstan D. E.; Zhang X. Do Stable Nanobubbles Exist in Mixtures of Organic Solvents and Water?. J. Phys. Chem. B 2010, 114, 6962–6967. 10.1021/jp911868j. 20438095

[ref31] Rak D.; Ovadova M.; Sedlak M. (Non)Existence of Bulk Nanobubbles: The Role of Ultrasonic Cavitation and Organic Solutes in Water. J. Phys. Chem. Lett. 2019, 10, 4215–4221. 10.1021/acs.jpclett.9b01402. 31295404

[ref32] Bunkin N. F.; Kochergin A. V.; Lobeyev A. V.; Ninham B. W.; Vinogradova O. I. Existence of charged submicrobubble clusters in polar liquids as revealed by correlation between optical cavitation and electrical conductivity. Colloids Surf. A Physicochem. 1996, 110, 207–212. 10.1016/0927-7757(95)03422-6.

[ref33] Cho S.-H.; Kim J.-Y.; Chun J.-H.; Kim J.-D. Ultrasonic formation of nanobubbles and their zeta-potentials in aqueous electrolyte and surfactant solutions. Colloids Surf. A Physicochem. 2005, 269, 28–34. 10.1016/j.colsurfa.2005.06.063.

[ref34] Uchida T.; Oshita S.; Ohmori M.; Tsuno T.; Soejima K.; Shinozaki S.; Take Y.; Mitsuda K. Transmission electron microscopic observations of nanobubbles and their capture of impurities in wastewater. Nanoscale Res. Lett. 2011, 6, 295 10.1186/1556-276X-6-295. 21711798 PMC3211361

[ref35] Duval E.; Adichtchev S.; Sirotkin S.; Mermet A. Long-lived submicrometric bubbles in very diluted alkali halide water solutions. Phys. Chem. Chem. Phys. 2012, 14, 4125–4132. 10.1039/c2cp22858k. 22337122

[ref36] Michailidi E. D.; Bomis G.; Varoutoglou A.; Kyzas G. Z.; Mitrikas G.; Mitropoulos A. C.; Efthimiadou E. K.; Favvas E. P. Bulk nanobubbles: Production and investigation of their formation/stability mechanism. J. Colloid Interface Sci. 2020, 564, 371–380. 10.1016/j.jcis.2019.12.093. 31918204

[ref37] Li M.; Ma X.; Eisener J.; Pfeiffer P.; Ohl C. D.; Sun C. How bulk nanobubbles are stable over a wide range of temperatures. J. Colloid Interface Sci. 2021, 596, 184–198. 10.1016/j.jcis.2021.03.064. 33845226

[ref38] Chae S. H.; Kim M. S.; Kim J. H.; Fortner J. D. Nanobubble Reactivity: Evaluating Hydroxyl Radical Generation (or Lack Thereof) under Ambient Conditions. ACS EST Engg. 2023, 3, 1504–1510. 10.1021/acsestengg.3c00124. PMC10581208 37854075

[ref39] Ma X.; Li M.; Xu X.; Sun C. On the role of surface charge and surface tension tuned by surfactant in stabilizing bulk nanobubbles. Appl. Surf. Sci. 2023, 608, 155232 10.1016/j.apsusc.2022.155232.

[ref40] Zhou Y.; Han Z.; He C.; Feng Q.; Wang K.; Wang Y.; Luo N.; Dodbiba G.; Wei Y.; Otsuki A.; Fujita T. Long-Term Stability of Different Kinds of Gas Nanobubbles in Deionized and Salt Water. Materials 2021, 14, 1808 10.3390/ma14071808. 33917489 PMC8038778

[ref41] Ushikubo F. Y.; Enari M.; Furukawa T.; Nakagawa R.; Makino Y.; Kawagoe Y.; Oshita S. Zeta-potential of Micro- and/or Nano-bubbles in Water Produced by Some Kinds of Gases. IFAC Proc. Vol. 2010, 43, 283–288. 10.3182/20101206-3-JP-3009.00050.

[ref42] Zhang H.; Guo Z.; Zhang X. Surface enrichment of ions leads to the stability of bulk nanobubbles. J. Soft Matter 2020, 16, 5470–5477. 10.1039/D0SM00116C. 32484196

[ref43] Ma X.; Li M.; Pfeiffer P.; Eisener J.; Ohl C. D.; Sun C. Ion adsorption stabilizes bulk nanobubbles. J. Colloid Interface Sci. 2022, 606, 1380–1394. 10.1016/j.jcis.2021.08.101. 34492474

[ref44] Satpute P. A.; Earthman J. C. Hydroxyl ion stabilization of bulk nanobubbles resulting from microbubble shrinkage. J. Colloid Interface Sci. 2021, 584, 449–455. 10.1016/j.jcis.2020.09.100. 33091868

[ref45] Takahashi M. ζ Potential of Microbubbles in Aqueous Solutions: Electrical Properties of the Gas–Water Interface. J. Phys. Chem. B 2005, 109, 21858–21864. 10.1021/jp0445270. 16853839

[ref46] Yildirim T.; Yaparatne S.; Graf J.; Garcia-Segura S.; Apul O. Electrostatic forces and higher order curvature terms of Young–Laplace equation on nanobubble stability in water. npj Clean Water 2022, 5, 18 10.1038/s41545-022-00163-4.

[ref47] Wang S.; Zhou L.; Gao Y. Can bulk nanobubbles be stabilized by electrostatic interaction?. Phys. Chem. Chem. Phys. 2021, 23, 16501–16505. 10.1039/D1CP01279G. 34286757

[ref48] Koshoridze S. I.; Levin Y. K. Comment on “Can bulk nanobubbles be stabilized by electrostatic interaction?” by S. Wang, L. Zhou and Y. Gao, *Phys. Chem. Chem. Phys.*, 2021, 23, 16501. Phys. Chem. Chem. Phys. 2022, 24, 10622–10625. 10.1039/D1CP01279G. 35437531

[ref49] Mopsik F. I. Dielectric Constant of N-Hexane as a Function of Temperature, Pressure, and Density. Journal of research of the National Bureau of Standards. Section A, Physics and chemistry 1967, 71a, 287–292. 10.6028/jres.071A.035. PMC6658454 31824054

[ref50] Albright P. S.; Gosting L. J. Dielectric Constants of the Methanol-Water System from 5 to 55°1. J. Am. Chem. Soc. 1946, 68, 1061–1063. 10.1021/ja01210a043. 20985620

[ref51] Khattab I. S.; Bandarkar F.; Fakhree M. A. A.; Jouyban A. Density, viscosity, and surface tension of water+ethanol mixtures from 293 to 323K. Korean Journal of Chemical Engineering 2012, 29, 812–817. 10.1007/s11814-011-0239-6.

[ref52] Thompson J. W.; Kaiser T. J.; Jorgenson J. W. Viscosity measurements of methanol–water and acetonitrile–water mixtures at pressures up to 3500bar using a novel capillary time-of-flight viscometer. Journal of Chromatography A 2006, 1134, 201–209. 10.1016/j.chroma.2006.09.006. 16996532

[ref53] Gregory A. P.; Clarke R. N. Traceable measurements of the static permittivity of dielectric reference liquids over the temperature range 5–50 °C. Measurement Science and Technology 2005, 16, 1506 10.1088/0957-0233/16/7/013.

[ref54] Cunningham G. P.; Vidulich G. A.; Kay R. L. Several properties of acetonitrile-water, acetonitrile-methanol, and ethylene carbonate-water systems. Journal of Chemical & Engineering Data 1967, 12, 336–337. 10.1021/je60034a013.

[ref55] Arce A.; Blanco A.; Soto A.; Vidal I. Densities, refractive indices, and excess molar volumes of the ternary systems water + methanol + 1-octanol and water + ethanol + 1-octanol and their binary mixtures at 298.15 K. Journal of Chemical & Engineering Data 1993, 38, 336–340. 10.1021/je00010a039.

[ref56] Sang B. H.; Jeon T.-I. Pressure-dependent refractive indices of gases by THz time-domain spectroscopy. Opt. Express 2016, 24, 29040–29047. 10.1364/OE.24.029040. 27958569

[ref57] Computational Chemistry Comparison and Benchmark DataBase. https://cccbdb.nist.gov/pollistx.asp (accessed 2023/12/4).

[ref58] Ahmed A. K. A.; Sun C.; Hua L.; Zhang Z.; Zhang Y.; Zhang W.; Marhaba T. Generation of nanobubbles by ceramic membrane filters: The dependence of bubble size and zeta potential on surface coating, pore size and injected gas pressure. Chemosphere 2018, 203, 327–335. 10.1016/j.chemosphere.2018.03.157. 29626810

[ref59] Nirmalkar N.; Pacek A. W.; Barigou M. Interpreting the interfacial and colloidal stability of bulk nanobubbles. J. Soft Matter 2018, 14, 9643–9656. 10.1039/C8SM01949E. 30457138

[ref60] Bui T. T.; Nguyen D. C.; Han M. Average size and zeta potential of nanobubbles in different reagent solutions. J. Nanopart. Res. 2019, 21, 173 10.1007/s11051-019-4618-y.

[ref61] Gauthier J. A.; Chen L. D.; Bajdich M.; Chan K. Implications of the fractional charge of hydroxide at the electrochemical interface. Phys. Chem. Chem. Phys. 2020, 22, 6964–6969. 10.1039/C9CP05952K. 32186292

[ref62] Marx D.; Tuckerman M. E.; Hutter J.; Parrinello M. The nature of the hydrated excess proton in water. Nature 1999, 397, 601–604. 10.1038/17579.

[ref63] Nirmalkar N.; Pacek A. W.; Barigou M. On the Existence and Stability of Bulk Nanobubbles. Langmuir 2018, 34, 10964–10973. 10.1021/acs.langmuir.8b01163. 30179016

[ref64] Meegoda J. N.; Hewage S. A.; Batagoda J. H. Application of the Diffused Double Layer Theory to Nanobubbles. Langmuir 2019, 35, 12100–12112. 10.1021/acs.langmuir.9b01443. 31433652

[ref65] Meegoda J. N.; Aluthgun Hewage S.; Batagoda J. H. Stability of Nanobubbles. Environ. Eng. Sci. 2018, 35, 1216–1227. 10.1089/ees.2018.0203.

[ref66] Zhang L.; Qiu Y.; Cheng L.; Wang Y.; Liu L.; Tu C.; Bowman D. C.; Burkey K. O.; Bian X.; Zhang W.; Hu S. Atmospheric CO_2_ Enrichment and Reactive Nitrogen Inputs Interactively Stimulate Soil Cation Losses and Acidification. Environ. Sci. Technol. 2018, 52, 6895–6902. 10.1021/acs.est.8b00495. 29771502

[ref67] Wang Q.; Zhao H.; Qi N.; Qin Y.; Zhang X.; Li Y. Generation and Stability of Size-Adjustable Bulk Nanobubbles Based on Periodic Pressure Change. Sci. Rep. 2019, 9, 1118 10.1038/s41598-018-38066-5. 30718777 PMC6362149

[ref68] Lee J. H.; Lee S. H.; Suh D. H. Using nanobubblized carbon dioxide for effective microextraction of heavy metals. J. CO2 Util. 2020, 39, 101163 10.1016/j.jcou.2020.101163.

[ref69] Antonio Cerron-Calle G.; Luna Magdaleno A.; Graf J. C.; Apul O. G.; Garcia-Segura S. Elucidating CO_2_ nanobubble interfacial reactivity and impacts on water chemistry. J. Colloid Interface Sci. 2022, 607, 720–728. 10.1016/j.jcis.2021.09.033. 34536932

[ref70] Wu D.; Zhang S.; Zhang H.; Zhang X.; Sun P. An experimental study on the characteristics of bulk nanobubbles generated by CO2 hydrate dissociation. Fuel 2022, 318, 123640 10.1016/j.fuel.2022.123640.

[ref71] Han Z.; Kurokawa H.; Matsui H.; He C.; Wang K.; Wei Y.; Dodbiba G.; Otsuki A.; Fujita T. Stability and Free Radical Production for CO2 and H2 in Air Nanobubbles in Ethanol Aqueous Solution. Nanomater. 2022, 12, 237 10.3390/nano12020237. PMC8779326 35055254

[ref72] Wang H.; Lawal T.; Achour S. H.; Sheng K.; Okuno R. Aqueous Nanobubble Dispersion of CO2 at Pressures Up To 208 bara. Energy Fuels 2023, 37, 19726–19737. 10.1021/acs.energyfuels.3c03660.

[ref73] Magdaleno A. L.; Cerrón-Calle G. A.; dos Santos A. J.; Lanza M. R. V.; Apul O. G.; Garcia-Segura S. Unlocking the Potential of Nanobubbles: Achieving Exceptional Gas Efficiency in Electrogeneration of Hydrogen Peroxide. Small 2024, 20, 2304547 10.1002/smll.202304547. 37621039

[ref74] Nirmalkar N.; Pacek A. W.; Barigou M. Bulk Nanobubbles from Acoustically Cavitated Aqueous Organic Solvent Mixtures. Langmuir 2019, 35, 2188–2195. 10.1021/acs.langmuir.8b03113. 30636423

[ref75] Jadhav A. J.; Barigou M. Proving and interpreting the spontaneous formation of bulk nanobubbles in aqueous organic solvent solutions: effects of solvent type and content. J. Soft Matter 2020, 16, 4502–4511. 10.1039/D0SM00111B. 32342965

[ref76] Ji Y.; Guo Z.; Tan T.; Wang Y.; Zhang L.; Hu J.; Zhang Y. Generating Bulk Nanobubbles in Alcohol Systems. ACS Omega 2021, 6, 2873–2881. 10.1021/acsomega.0c05222. 33553905 PMC7860054

[ref77] Sharma H.; Trivedi M.; Nirmalkar N. Do Nanobubbles Exist in Pure Alcohol?. Langmuir 2024, 40, 1534–1543. 10.1021/acs.langmuir.3c03592. 38176064

[ref78] Alheshibri M.; Craig V. S. J. Generation of nanoparticles upon mixing ethanol and water; Nanobubbles or Not?. J. Colloid Interface Sci. 2019, 542, 136–143. 10.1016/j.jcis.2019.01.134. 30735888

[ref79] Chen N.; Wen Z.; Li X.; Ye Z.; Ren D.; Xu J.; Chen Q.; Ma S. Controllable preparation and formation mechanism of monodispersed bulk nanobubbles in dilute ethanol-water solutions. Colloids Surf. A Physicochem. 2021, 616, 126372 10.1016/j.colsurfa.2021.126372.

[ref80] Liu X.-L.; Mao M.; Ren M.-G.; Tong Y.; Song Q.-H. Fluoride-sensing properties tuning of donor-substituted triarylboranes through varying donors: The fluorescent mode, the fluoride-binding affinity and the selectivity. Sens. Actuators B Chem. 2014, 200, 317–324. 10.1016/j.snb.2014.04.070.

[ref81] Karimi M.; Gabbaï F. P. Hydrogen Bond-Assisted Fluoride Binding by a Stiborane. Z. Anorg. Allg. Chem. 2022, 648, e202200098 10.1002/zaac.202200098.

