# Supplementary material for: Polarizing Perspectives: Ion- and Dipole-Induced Dipole Interactions Dictate Bulk Nanobubble Stability
Source: J Phys Chem B. 2024 Jul 11;128(29):7263–70. doi: 10.1021/acs.jpcb.4c03973 (PMC11284786; doi:10.1021/acs.jpcb.4c03973)
Supplement: Supplementary file 1 — jp4c03973_si_001.pdf [file jp4c03973_si_001.pdf]

Polarizing Perspectives: Ion- and Dipole-Induced Dipole Interactions Dictate Bulk Nanobubble Stability

Mohammadjavad Karimi, Gholamabbas Parsafar, Hamidreza Samouei*
Department of Petroleum Engineering, Texas A&M University, College Station, TX 77843, USA
Email address: samouei@tamu.edu

Supporting Information

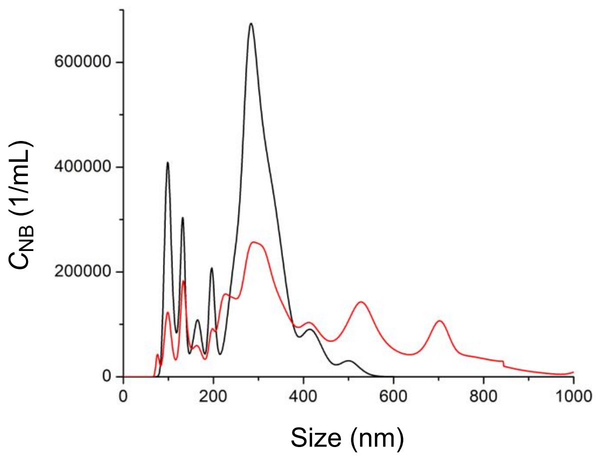

Figure S1. Particle size distribution of NBs of He in water 1 hour (black) and 1 week (red) after NB generation.

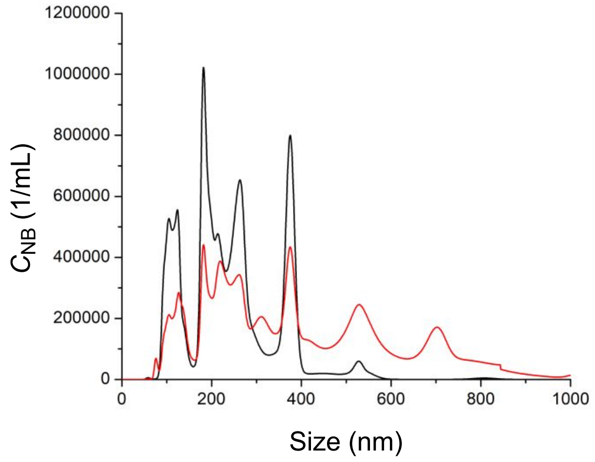

Figure S2. Particle size distribution of NBs of O_2 in water 1 hour (black) and 1 week (red) after NB generation.

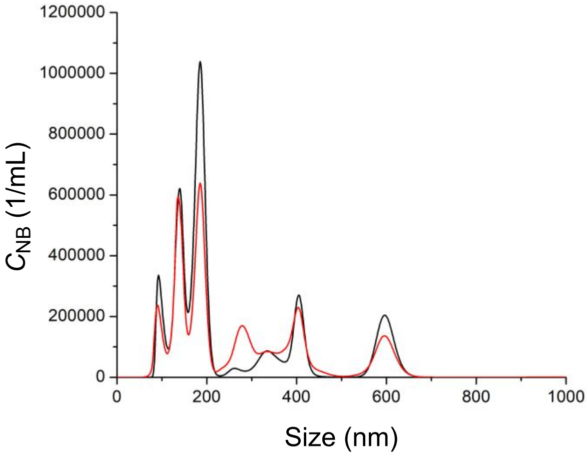

Figure S3. Particle size distribution of NBs of N_2 in water 1 hour (black) and 1 week (red) after NB generation.

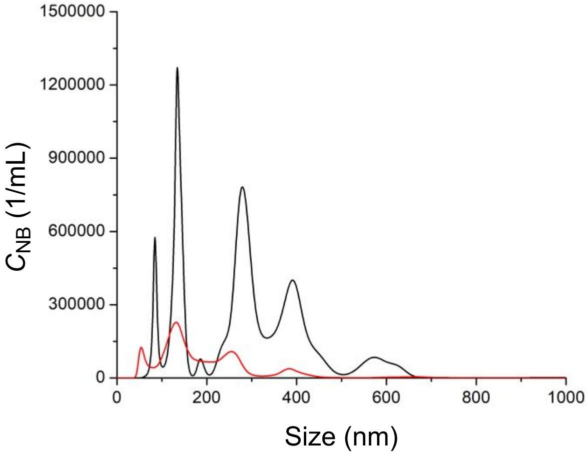

Figure S4. Particle size distribution of NBs of CO_2 in water 1 hour (black) and 1 week (red) after NB generation.

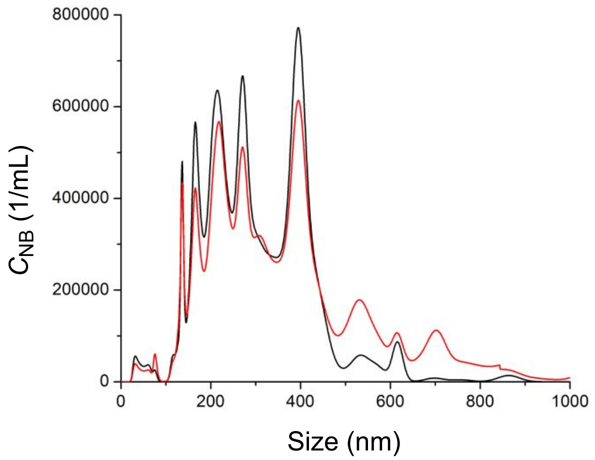

Figure S5. Particle size distribution of NBs of n-butane in water 1 hour (black) and 1 week (red) after NB generation.

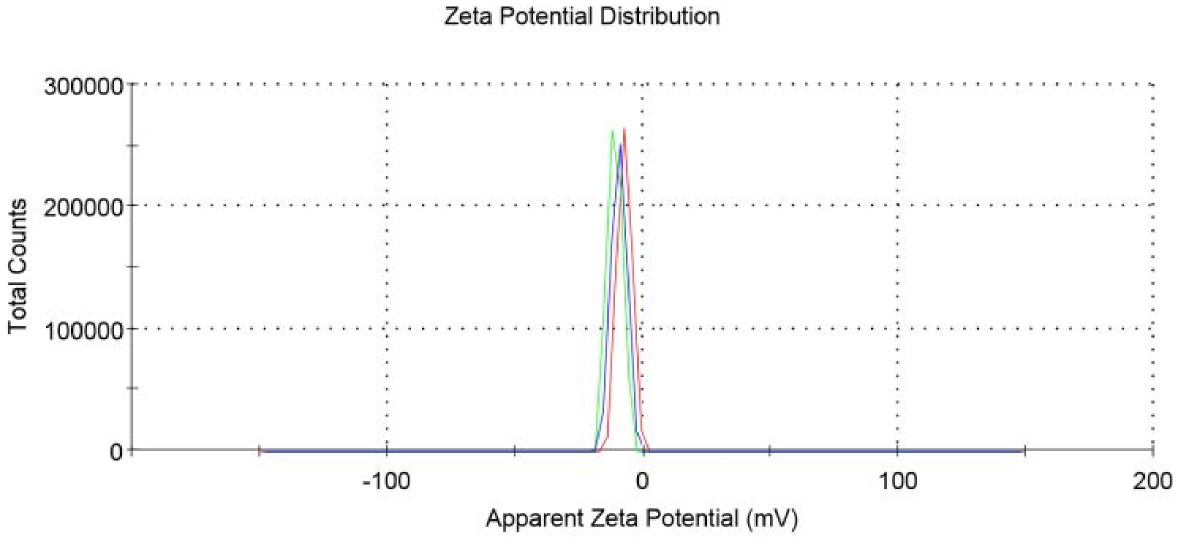

Figure S6. Zeta potential distribution of NBs of helium in water 1 hour after NB generation.

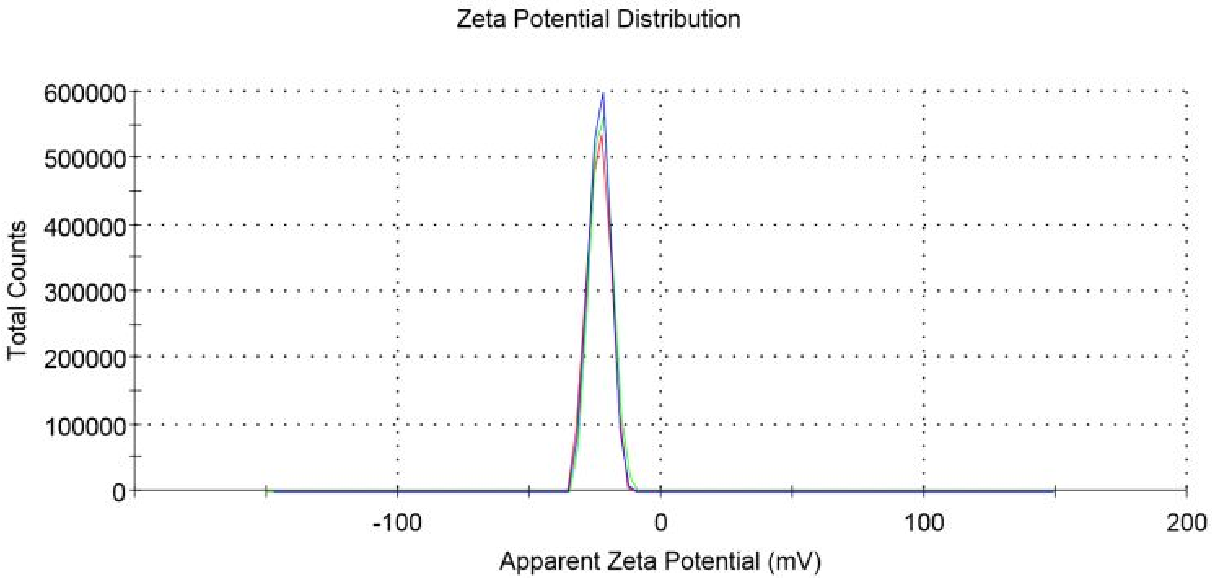

Figure S7. Zeta potential distribution of NBs of O_2 in water 1 hour after NB generation.

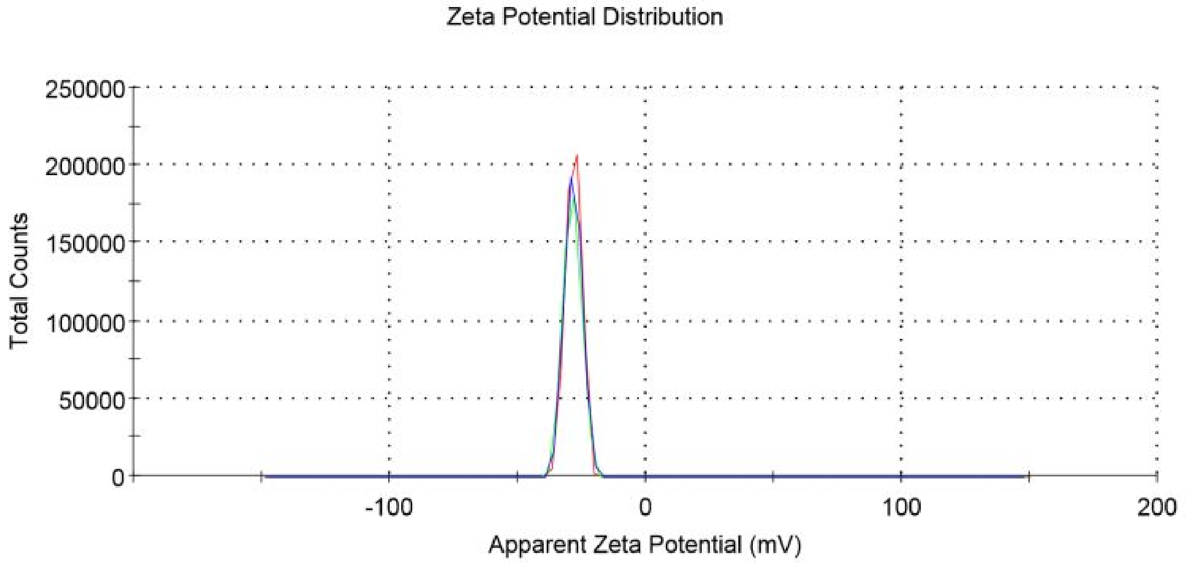

Figure S8. Zeta potential distribution of NBs of N_2 in water 1 hour after NB generation.

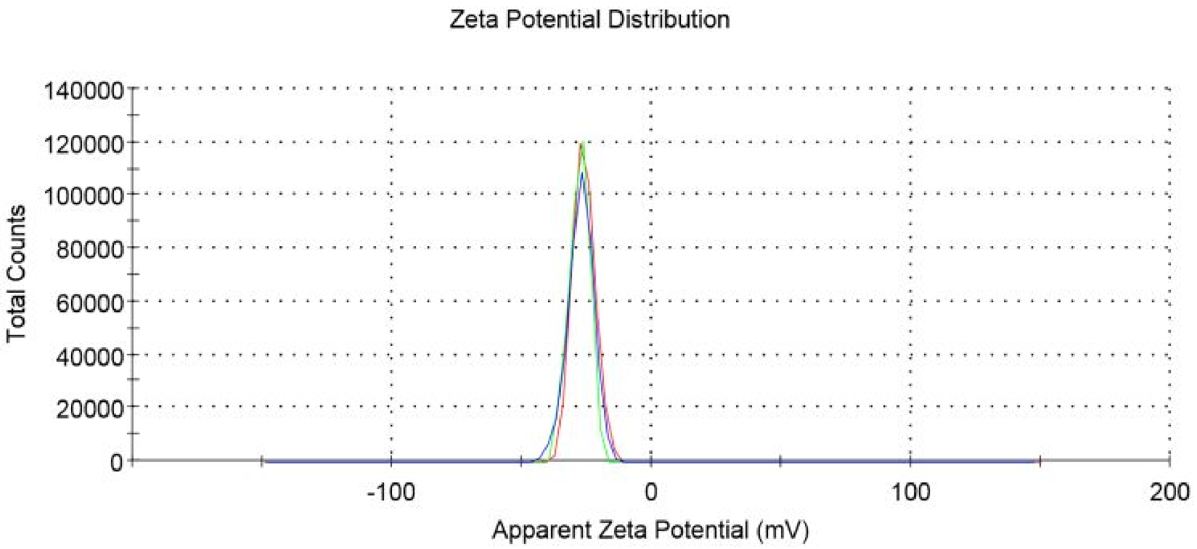

Figure S9. Zeta potential distribution of NBs of CO_2 in water 1 hour after NB generation.

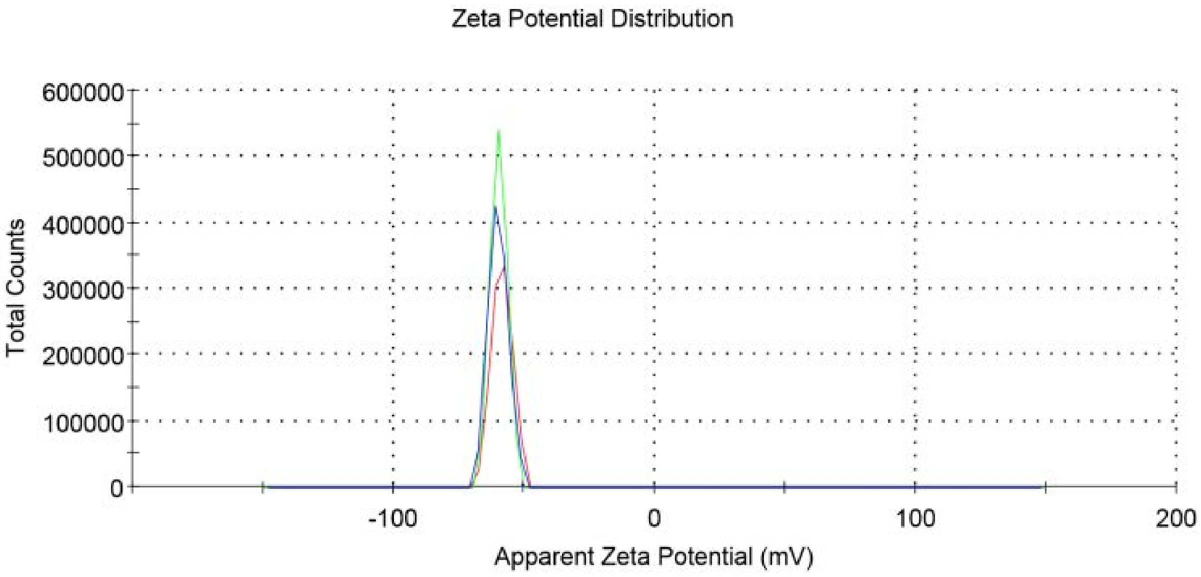

Figure S10. Zeta potential distribution of NBs of n-butane in water 1 hour after NB generation.

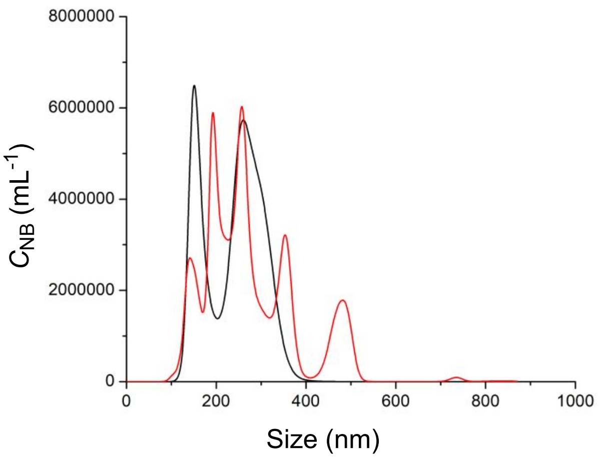

Figure S11. Particle size distribution of NBs of N_2 in ethanol 1 hour (black) and 1 day (red) after NB generation.

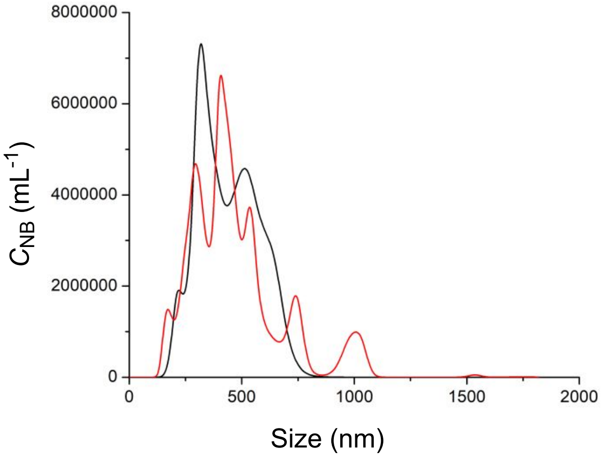

Figure S12. Particle size distribution of NBs of N_2 in methanol 1 hour (black) and 1 day (red) after NB generation.

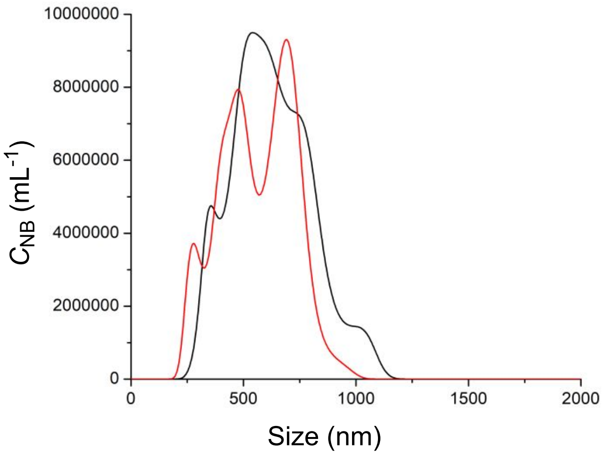

Figure S13. Particle size distribution of NBs of N_2 in acetonitrile 1 hour (black) and 1 day (red) after NB generation.
